# Letter to the Editor: Examining pregnancy outcomes in alcohol-associated hepatitis: A call for further research

**DOI:** 10.1097/HC9.0000000000000723

**Published:** 2025-06-19

**Authors:** Mohamed El-Kassas, Mohamed Elbadry, Khalid Alswat

**Affiliations:** 1 Faculty of Medicine, Department of Endemic Medicine, Helwan University, Cairo, Egypt; 2 Department of Medicine, Liver Disease Research Center, College of Medicine, King Saud University, Riyadh, Saudi Arabia

To the editor,

We have read with great interest the article by Cooper et al,^1^ titled “Pregnancy and liver-related outcomes after alcohol-associated hepatitis: A global multicenter study,” published in *Hepatology Communications*. This study provides valuable insights into liver-related outcomes in women with alcohol-associated hepatitis (AH), an increasingly relevant topic given the rising incidence of AH among young women. These findings contribute significantly to our understanding of the interplay between women’s reproductive health and liver disease. However, several aspects of this study warrant further exploration to enhance the robustness and applicability of the findings. The retrospective design of this study, which is useful for hypothesis generation, inherently limits the ability to establish causality as it is subject to selection bias, unmeasured confounding, and potential inaccuracies in data capture. Although propensity score matching was employed to balance key demographic factors, residual confounding from unmeasured variables such as socioeconomic status, nutrition, access to health care, and variations in alcohol consumption may still impact both pregnancy- and liver-related outcomes.^2^ Addressing these limitations in future prospective studies could provide a more comprehensive understanding of these associations. Another critical consideration is the reliance on the International Classification of Diseases, Tenth Revision codes for diagnosing AH and defining pregnancy-related outcomes. Although recent updates in administrative databases using ICD codes facilitate large-scale analyses, they introduce the potential for misclassification bias, particularly given the clinical variability in defining AH.^3^ A more granular approach, incorporating detailed laboratory and clinical data, would strengthen the validity of the findings.

Furthermore, the study lacks detailed information on patterns of alcohol consumption, a crucial determinant of AH severity, and pregnancy outcomes. Analyzing data related to the frequency, quantity, and duration of alcohol use could provide a more nuanced understanding of the effects of alcohol on pregnancy outcomes.

The proposed link between systemic inflammation and adverse pregnancy outcomes, particularly preeclampsia, is an intriguing hypothesis. However, the study could not collect data related to inflammatory markers such as IL6 and TNF-α, which are key mediators in both AH and hypertensive disorders during pregnancy.^4^ Future research integrating biomarker analyses could help elucidate the underlying pathophysiological mechanisms.

The generalizability of the results is another area of consideration. The study cohort was predominantly Caucasian (69.3%) and non-Hispanic (74.5%), which may limit the applicability of our findings to more diverse populations. Given the known racial and ethnic disparities in both AH and pregnancy outcomes, ensuring a broader representation in future studies would enhance the external validity of the conclusions. Finally, the role of medication-assisted therapy in mitigating adverse outcomes among women with AH deserves further exploration. While the study mentioned medication-assisted therapy use, a more detailed analysis of its impact on pregnancy and liver-related outcomes would be valuable for guiding clinical management.^5^

The study by Cooper et al contributed significantly to the understanding of AH in pregnancy. Addressing these considerations through prospective studies with detailed alcohol consumption data, biomarker analyses, and socioeconomic adjustments will further clarify these critical relationships and refine management strategies for this high-risk population.
